# Physiologic Scoring Systems versus Glasgow Coma Scale in Predicting In-Hospital Mortality of Trauma Patients; a Diagnostic Accuracy Study

**DOI:** 10.22037/aaem.v9i1.1376

**Published:** 2021-09-23

**Authors:** Farhad Heydari, Reza Azizkhani, Omid Ahmadi, Saeed Majidinejad, Mohammad Nasr-Esfahani, Ahmad Ahmadi

**Affiliations:** 1 Department of Emergency Medicine, Faculty of Medicine, Isfahan University of Medical Sciences, Isfahan, Iran.

**Keywords:** Multiple trauma, Injury severity score, scoring system/ Clinical Decision Rules, Emergency service, hospital, Patient outcome assessment, Prognosis

## Abstract

**Introduction:**

In recent years, several scoring systems have been developed to assess the severity of trauma and predict the outcome of trauma patients. This study aimed to compare Rapid Emergency Medicine Score (REMS), Modified Early Warning Score (MEWS), Injury Severity Score (ISS), and Glasgow Coma Scale (GCS) in predicting the in-hospital mortality of trauma patients.

**Methods:**

This diagnostic accuracy study was done on adult patients admitted to the emergency department (ED) between June 21, 2019, and September 21, 2020, following multiple trauma. Patients were followed as long as they were hospitalized. The REMS, MEWS, GCS, and ISS were calculated after data gathering and comprehensive assessment of injuries. Receiver operating characteristics (ROC) analysis was performed to examine the prognostic performance of the four different tools.

**Results:**

Of the 754 patients, 32 patients (4.2%) died and 722 (95.8%) survived after 24 hours of admission. The mean age of the patients was 38.54 ± 18.58 years (78.9% male). The area under the ROC curves (AUC) of REMS, MEWS, ISS, and GCS score for predicting in-hospital mortality were 0.942 (95% CI [0.923-0.958]), 0.886 (95% CI [0.861-0.908]), 0.866 (95% CI [0.839-0.889]), and 0.851 (95% CI [0.823-0.876]), respectively. The AUC of REMS was significantly higher than GCS (p=0.035). The sensitivities of GCS ≤ 11, ISS ≥ 13, REMS ≥ 4, and MEWS ≥ 3 scores for in-hospital mortality were 0.56, 0.97, 0.81, and 0.94, respectively. Also, the specificities of GCS, ISS, REMS, and MEWS scores for in-hospital mortality were 0.93, 0.82, 0.81, and 0.85, respectively.

**Conclusion:**

It seems that REMS is more accurate than GCS, ISS, and MEWS in predicting in-hospital mortality ≥ 24 hours of multiple trauma patients.

## 1. Introduction

Trauma and unintentional injuries kill more than 175,000 Americans each year and are the leading cause of death in people under 45 years of age (1). Also, trauma causes severe complications, disability, and financial and social costs (2, 3). Early diagnosis and appropriate triage and immediate treatment decrease in-hospital mortality and are cost-effective (4). In recent years, several scoring systems have been implemented to assess the severity of the injuries and determine which patients need intensive observation, treatment, and appropriate allocation of healthcare resources (3, 5, 6). National Early Warning Score (NEWS), Rapid Acute Physiology Score (RAPS), Rapid Emergency Medicine Score (REMS), Worthing Physiological Scoring System (WPSS), and Modified Early Warning Score (MEWS) are some of the most commonly used scoring systems.

Glasgow Coma Scale (GCS) is used to assess a person's level of consciousness and head injury severity. This scale is used by emergency medical services, nurses, and physicians, and is applied for all acute medical and trauma patients (7). 

Injury Severity Score (ISS) is an established medical score to assess trauma severity. It is an anatomy-based scoring system to predict the outcome of victims with multiple injuries (3). 

REMS, which is a powerful predictor of in-hospital mortality among medical (non-trauma) patients admitted to the hospital was developed in 2004 (8). REMS consists of six key parameters: patient’s age, mean arterial pressure (MAP), heart rate, respiratory rate, SpO2, and Glasgow Coma Scale.

Also, MEWS can be used to identify patients who are at risk of clinical deterioration and who may require a higher level of care (9). MEWS comprises five physiological parameters: systolic blood pressure (SBP), Heart rate, respiratory rate, temperature, and AVPU Score.

This study aimed to compare the diagnostic accuracy of 3 physiologic scoring systems including Rapid Emergency Medicine Score (REMS), Modified Early Warning Score (MEWS), and Injury Severity Score (ISS), as well as Glasgow Coma Scale (GCS) in predicting in-hospital mortality of trauma patients.

## 2. Methods

***2.1. Study design and setting***

This was a prospective diagnostic accuracy study of adult multiple trauma patients admitted to Al-Zahra and Kashani Hospitals, two university educational hospitals, affiliated with Isfahan University of Medical Sciences, Isfahan, Iran. This study was approved by the Ethics Committee of Isfahan University of Medical Sciences (IR.MUI.MED.REC.1398.340) and an informed consent form was obtained from patients.

***2.2. Participants***

All multiple trauma patients (two or more body region injuries), who were aged 18 years and older and were admitted to the emergency department (ED) between June 21, 2019, and September 21, 2020, were included in the study. Patients were enrolled regardless of trauma severity. Exclusion criteria were patients with missing data necessary to calculate scores, discharge or death in less than 24 hours from admission, patients transferred from other hospitals, burn or drowning-related injuries, pregnancy, and discharge against medical advice.

ISS was calculated after complete evaluation of the patient and receiving the results such as imaging results, intervention findings, and operative records, so comprehensive assessment of injuries could take substantial time, therefore hospitalization for at least 24 hours was considered to calculate ISS in all patients.

***2.3. Data gathering***

After multiple trauma patients arrived at the ED, the triage nurse evaluated them on the basis of Emergency Severity Index (ESI) version 4, and then, the patients were transferred to the emergency room according to the level of the triage. Then all participants were examined by emergency medicine residents upon their arrival and they took over the patient’s treatment and follow-up. Sampling was performed using the convenience method.

Age, sex, systolic blood pressure (SBP), diastolic blood pressure (DBP), respiratory rate (RR), heart rate (HR), GCS, AVPU score, temperature, oxygen saturation, length of hospital stay, mechanism of injury, triage level based on ESI and in-hospital mortality, were collected for each patient. Patients were followed during their hospital stay to evaluate their final outcome.  The in-hospital mortality was defined as death during the present hospital stay. 

REMS consists of 6 parameters, 5 physiological and 1 age (8). The highest score is 26 with higher values being indicative of a worse prognosis. MEWS consists of 5 physiological parameters (9). The range of MEWS total score is from 0 to a maximum of 14. ISS is an anatomical scoring system for patients with multiple injuries. ISS is based on Abbreviated Injury Scale (AIS), which divides the body into six regions. ISS is calculated as the sum of the squares of the highest AIS code in each of the three most severely injured body regions and has a range from 0 to 75 (3). REMS and MEWS scores were calculated according to the physiological criteria that were evaluated on admission to ED. ISS scores were calculated after data gathering and comprehensive assessment of injuries.

***2.4. Statistical analysis***

Considering the 5.2% prevalence of in-hospital mortality in trauma patients (10) and area under the curve of GCS in predicting in-hospital mortality being 0.88 (11) and marginal error of 0.05, the minimum required sample size was calculated to be 337 patients. SPSS version 25.0 (IBM, Armonk, NY) was used to analyze the variables. Categorical variables were described as frequency and percentage and continuous variables were described as mean and standard deviation (SD) or median and interquartile ranges (IQR). Chi-square or Fisher’s exact test were used for the comparisons between categorical variables and independent samples t-test or Mann-Whitney U test were used for the comparisons between continuous variables.

The predictive values of REMS, MEWS, ISS, and GCS in predicting in-hospital mortality were compared using the area under the receiver operating characteristic curve (AUC) with a 95% confidence interval (CI). Sensitivity, specificity, positive and negative likelihood ratios, and positive and negative predictive values with 95% CI were reported for each score. P-values less than 0.05 were considered statistically significant.

## 3. Results

**Figure 1:**
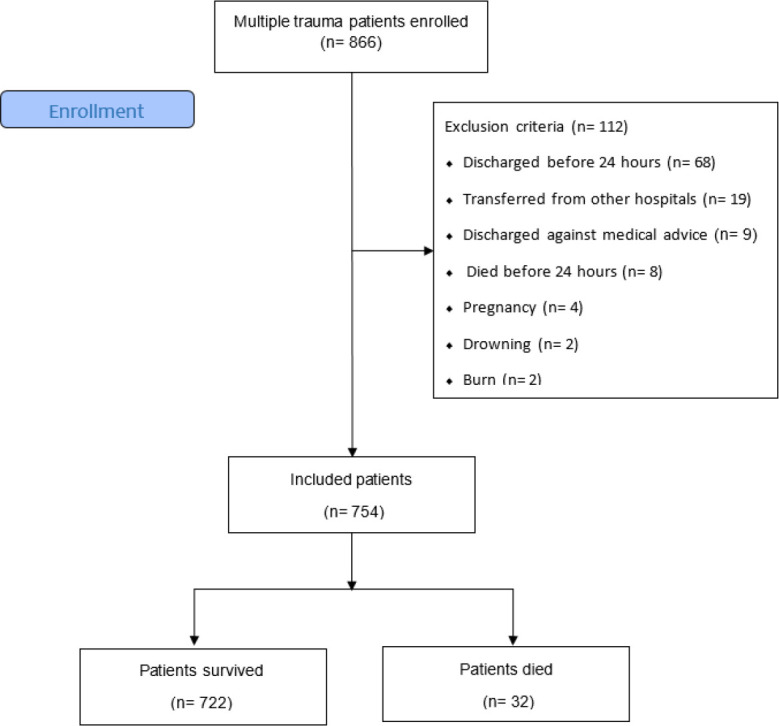
CONSORT Flow Diagram

Of the 754 patients included in this study, 32 patients (4.2%) died and 722 patients (95.8%) were discharged from hospital (Figure 1). The mean age of the patients was 38.54 ± 18.58 (18 –94) years (78.9% male). Road injuries were the main cause of trauma (70.3%) followed by falls (16.5%). 391 patients (51.9%) required surgery and 185 patients (24.5%) were admitted to the ICU.

The median GCS, ISS, REMS, and MEWS scores (IQR) were 15 (14-15), 9 (5-14), 0 (0-3) and 1 (1-2), respectively. According to the emergency severity index (ESI) triage system, 21.0%, 52.5%, and 26.5% of the patients were categorized as levels I, II, and III, respectively. The mean duration of hospital stay was 6.28 ± 5.78 days. Mean vital sign measures of the patients and other baseline characteristics have been reported in table 1.

**Figure 2:**
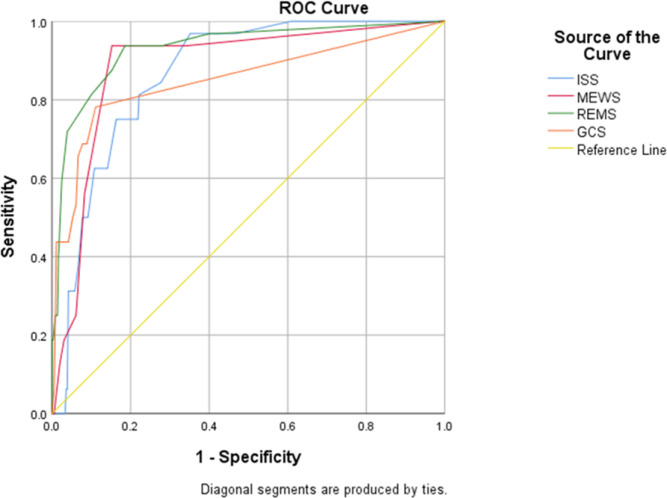
Receiver operating characteristic (ROC) curves of Injury Severity Score (ISS); Glasgow Coma Scale (GCS); Modiﬁed Early Warning Score (MEWS); and Rapid Emergency Medicine Score (REMS) for predicting in-hospital mortality (≥ 24 hours) of multiple trauma patients

The area under the ROC curves of REMS, MEWS, ISS, and GCS scores in predicting the in-hospital mortality of trauma patients were 0.942 (95% CI: 0.923-0.958), 0.886 (95% CI: 0.861-0.908), 0.866 (95% CI: 0.839-0.889) and 0.851 (95% CI: 0.823-0.876), respectively (figure 2). The optimal cut-off values for the mentioned scores were ≥4 for REMS, ≥3 for MEWS score, ≥13 for ISS, and ≤11 for GCS. The sensitivities of GCS, ISS, REMS, and MEWS scores in these cutoff points were 0.56, 0.97, 0.81, and 0.94, respectively. Also, the specificities of GCS, ISS, REMS, and MEWS scores for in-hospital mortality were 0.93, 0.82, 0.81, and 0.85, respectively (Table 2).

GCS was similar to MEWS (p=0.456) and ISS (p=0.723) in predicting in-hospital mortality. However, REMS was significantly better than GCS (p=0.035) in predicting in-hospital mortality (Table 3).

## 4. Discussion

Based on the results of this study, REMS was better than MEWS, ISS, and GCS in predicting in-hospital mortality occurring ≥ 24 hours after admission among adult multiple trauma patients referring to ED. Based on calculated AUCs, the results showed REMS was an excellent predictor of in-hospital mortality (AUC = 0.94), and MEWS, ISS, and GCS were good predictors of in-hospital mortality (AUC = 0.89, 0.87, and, 0.85). 

Despite advances in injury prevention and medical care, trauma deaths remain a major public health problem worldwide. To improve overall survival and management outcomes, it is important to quickly and accurately determine the severity of trauma in patients admitted to the ED. Various scoring systems have been developed for the classification of injuries, which include physiologic and anatomic systems (12).

Each of these systems has its specific limitations and advantages, but an efficient scoring system should have fewer variables, be easy to use and be accurate, especially in emergency settings. One of the oldest trauma scores is ISS. Several studies have shown that ISS is a valid predictor of in-hospital mortality (3, 5, 6, 8, 9). One of the important limitations of ISS is the inability to be calculated in the initial evaluation of the patient. ISS can be calculated after a comprehensive assessment of the patient and identification of all injuries.

Several scoring systems have been developed to objectively measure the initial condition of a trauma patient, and these may also serve as prognostic indicators for specific patients (9-12). REMS and MEWS have acceptable predictive values for in-hospital mortality and are good choices for use in emergency settings.

It seems that REMS and MEWS scores are superior to other predictors because they both include vital signs (e.g., SBP and RR) and neurological variables (e.g., AVPU, Motor, and Speech), which are strongly related to mortality risk. AUCs of REMS and MEWS were more than GCS, this indicates that adding parameters such as BP, HR, RR, O2 saturation, and body temperature to the level of consciousness, which is usually assessed using GCS, increases the efficiency of GCS in predicting the outcomes of traumatic patients. Some of the REMS and MEWS parameters (MAP or SBP, GCS or AVPU and HR) were significantly associated with mortality risk, while age, oxygen saturation, temperature and RR were independent predictors of in-hospital mortality.

In most previous studies, REMS has been used to predict mortality in non-surgical patients. In the study conducted by Olsson et al., the REMS was found to be a strong predictor of both in-hospital and long-term mortality in non-surgical patients in the ED (8). Goodacre et al. compared REMS and RAPS scores in predicting in-hospital mortality of 5583 patients who were brought by the emergency ambulance and hospitalized. They found that REMS is effective in predicting mortality among medical patients (13). 

REMS can be rapidly determined in 20 minutes and has been shown to be compatible with mortality rate prediction in patients with trauma in previous studies. Imholff et al. showed that a higher REMS score is associated with an increase in the mortality rate of trauma patients (10). Nakhjavan-Shahraki et al. suggested that REMS could be used to predict mortality (AUC=0.93) and poor outcomes (p=0.001) in patients with trauma in emergency settings (14). The findings of the current study are consistent with those of the previous studies, which found that REMS is a simple and accurate predictor of in-hospital mortality for multiple trauma patients.

MEWS has been used to initially identify the risk of mortality and to predict the clinical outcomes of patients (15-17). Several studies showed that MEWS is useful in predicting the severity of trauma among patients. In a previous study, MEWS was a fair predictor of in-hospital mortality (AUC, 0.79; 95% CI, 0.74-0.83) (18). In contrast, in another study, MEWS was a good predictor of in-hospital mortality (AUC, 0.90; 95% CI, 0.88-0.92) in trauma patients (18). Consistently, our results showed that MEWS is a good predictor (AUC=0.89) of in-hospital mortality in multiple trauma patients.

Bulut et al. reported that the prognostic value of REMS model for mortality of medical and surgical patients referring to EDs was significantly higher than = MEWS (9). The results of the current study showed that REMS is superior to MEWS and GCS in predicting in-hospital mortality for trauma patients. Although the sensitivity of REMS ≥ 4 and MEWS ≥ 3 (96.87% and 93.75) in predicting in-hospital mortality were higher than GCS ≤ 11, the specificity of GCS (93.35%) was higher than other scores. When specificity is high, it is less likely to give a false-positive. On the other hand, the sensitivity of GCS was only 56.25 %, which means that there are many false-negative results. In serious and life threatening conditions we should use tests or methods with high sensitivity to decrease false negative rates. Also, in the present study, the PPV was reported to be low and the NPV was reported to be high. This could be due to the low prevalence of in-hospital mortality. In this study, in-hospital mortality was 4.2%. PPV and NPV are directly related to prevalence.

**Table 1:** Comparison of demographic and clinical characteristics of multiple trauma patients according to in-hospital mortality after ≥ 24 hours of admission

**Characteristics**	**Total ** **(n=754)**	**Survived ** **(n=722)**	**Non-Survived** **(n=32)**	**P value**
**Age, (year)**				
Mean ± SD	38.54±18.58	38.13±18.09	47.75±28.31	0.133^1^
**Sex, n(%)**				
Female	159 (21.1)	155 (21.5)	4 (12.5)	0.223^2^
Male	595 (78.9)	567 (78.5)	28 (87.5)	
**Mechanism, n (%)**				
Road injuries	529 (70.3)	507 (70.3)	22 (68.7)	0.116^2^
Fall	124 (16.5)	114 (15.8)	10 (31.3)	
Assault	95 (12.7)	95 (13.1)	0 (0.0)	
Others	6 (0.5)	6 (0.8)	0 (0.0)	
**Triage level, n (%)**				
1	158 (21.0)	130 (18.0)	28 (87.5)	<0.001^2^
2	396 (52.5)	392 (54.3)	4 (12.5)	
3	200 (26.5)	200 (27.7)	0 (0.0)	
**Glasgow coma scale, n (%)**				
3-8	40 (5.3)	20 (2.8%)	20 (62.5%)	<0.001^2^
9-12	30 (4.0)	26 (3.6%)	4 (12.5%)	
13-14	24 (3.2)	24 (3.3%)	0 (0.0%)	
15	660 (87.5)	652 (90.3%)	8 (25.0%)	
**Length of stay, (day)**				
Mean ± SD	6.25±5.78	6.31±5.84	5.14±5.11	0.625^1^
Vital signs*				
HR, (bpm)	87.42±14.19	87.03±13.74	96.24±20.45	0.014^1^
SBP, (mmHg)	129.87±19.31	130.34±16.87	119.18±17.45	<0.001^1^
MAP, (mmHg)	90.23±13.13	90.85±32.70	76.15±11.48	<0.001^1^
RR, (bpm)	19.20±3.68	19.18±3.48	19.68±7.54	0.785^1^
Temp, (°c)	36.97±0.31	36.99±0.31	36.88±0.16	0.597^1^
O2 SAT, (%)	94.58±3.11	94.57±2.99	94.88±5.03	0.5841
Injury severity (median (IQR))*				
ISS	9 (5-14)	9 (5-14)	23 (15-29)	<0.001^3^
GCS	15 (14-15)	15 (14-15)	11 (4-15)	<0.001^3^
MEWS	1 (1-2)	1 (1-2)	4 (3-4.75)	<0.001^3^
REMS	0 (0-3)	0 (0-3)	8 (6-10.5)	<0.001^3^

IQR: Interquartile range, SD: standard deviation, HR: Heart Rate; SBP: Systolic Blood Pressure; MAP: Mean Arterial Pressure; RR: Respiratory Rate; Temp: Temperature; SAT: saturation; ISS: Injury Severity Score; GCS: Glasgow Coma Scale; MEWS: Modiﬁed Early Warning Score; REMS: Rapid Emergency Medicine Score. ^ 1^ Analyzed using via independent-samples t test. ^2^ Analyzed using Fisher's exact test. ^3^ Analyzed using Mann-Whitney U test. * These data were evaluated at the time of admission to emergency department

**Table 2:** Screening performance characteristics of physiologic scoring systems (REMS, MEWS, ISS) and Glasgow coma scale (GCS) in prediction of in-hospital mortality

**GCS**	**ISS**	**MEWS**	**REMS**	**Variables**
≤ 11	≥13	≥3	≥4	**Cut-off**
56.25 (37.7 - 73.6)	81.25 (54.4-96.0)	93.75 (79.2 - 99.2)	96.87 (83.8 - 99.9)	**Sensitivity **
93.35 (91.3 - 95.1)	81.59 (77.1-85.5)	84.76 (81.9 - 87.3)	81.30 (78.3 - 84.1)	**Specificity **
37.0 (19.4-57.6)	16.7 (9.2-26.8)	21.3 (11.9-33.7)	16.2 (8.7-26.6)	**PPV**
98.2 (96.2-99.4)	99.0 (97.0-99.8)	99.0 (97.2-99.8)	98.6 (96.6-99.6)	**NPV**
8.46 (5.6 - 12.7)	4.41 (3.2-6.1)	6.15 (5.1 - 7.5)	5.18 (4.4 - 6.1)	**PLR **
0.47 (0.3 - 0.7)	0.23 (0.08-0.6)	0.07 (0.02 - 0.3)	0.04 (0.01 - 0.3)	**NLR **
0.851 (0.823-0.876)	0.866 (0.839-0.889)	0.886 (0.861-0.908)	0.942 (0.923-0.958)	**AUC **

Data are presented with 95% confidence interval. Abbreviations: REMS: Rapid Emergency Medicine Score; MEWS: Modiﬁed Early Warning Score; ISS: Injury Severity Score; GCS: Glasgow Coma Scale; PPV: Positive predictive value; NPV: Negative predictive value; AUC; Area Under Curve; PLR: Positive Likelihood Ratio, NLR: Negative Likelihood Ratio.

**Table 3:** Comparison of the area under the receiver operating characteristic (ROC) curve of studied scores

**Scores**	**REMS**	**MEWS**	**GCS**	**ISS**
REMS		0.107	** 0.035**	**0.010**
MEWS			0.456	0.528
GCS				0.723
ISS				

ISS: Injury Severity Score; GCS: Glasgow Coma Scale; MEWS: Modiﬁed Early Warning Score; REMS: Rapid Emergency Medicine Score.

## 5. Limitations

Our study has some limitations. First, convenience sampling method was used and the researcher was present in the ED, which may have caused selection bias. Second, patients who died in less than 24 hours and those who died upon arrival were excluded; the lack of information on these patients may have caused a spectrum bias.

## 6. Conclusion:

The findings of this study revealed that REMS is an excellent predictor of in-hospital mortality ≥ 24 hours after admission and MEWS, GCS, and ISS are good alternatives for predicting in-hospital mortality in multiple trauma patients. 

## 7. Declarations

### 7.1 Author contributions

F.H., S.M., A.A., M.N.E., and R.A. contributed to the conception, study design, and data collection and evaluation. F.H., R.A., and A.A. contributed to statistical analysis, and interpretation of data. F.H. and R.A. were responsible for overall supervision. F.H., A.A., and R.A. drafted the manuscript, which was revised by M.N.E., and S.M. All authors performed editing and approving the final version of this paper for submission, also participated in the finalization of the manuscript and approved the final draft.

### 7.2. Acknowledgments

The authors would like to express their gratitude to the staff of the EDs of Al-Zahra and Kashani Hospitals, Isfahan, Iran. 

### 7.3. Conflict of Interests

The authors declare no conflict of interest.

### 7.4. Funding

This study was conducted with the support of Isfahan University of Medical Sciences. 
